# Highly Flexible Triboelectric Nanogenerator Using Porous Carbon Nanotube Composites

**DOI:** 10.3390/polym15051135

**Published:** 2023-02-24

**Authors:** Jaehee Shin, Sungho Ji, Hanchul Cho, Jinhyoung Park

**Affiliations:** 1 Department of Mechatronics Engineering, Korea University of Technology & Education, 600, Chungjeol-ro, Byeongcheon-myeon, Dongnam-gu, Chungcheongnam-do, Cheonan-si 31253, Republic of Korea; 2 Precision Mechanical Process and Control R&D Group, Korea Institute of Industrial Technology (KITECH), 42-7, Baegyang-daero 804 beon-gil, Sasang-gu, Busan 46938, Republic of Korea

**Keywords:** TENG, conductive sponge, CNTs, silicone rubber, energy harvesting, flexible device

## Abstract

The rapid development of portable and wearable electronic devices has led researchers to actively study triboelectric nanogenerators (TENGs) that can provide self-powering capabilities. In this study, we propose a highly flexible and stretchable sponge-type TENG, named flexible conductive sponge triboelectric nanogenerator (FCS-TENG), which consists of a porous structure manufactured by inserting carbon nanotubes (CNTs) into silicon rubber using sugar particles. Nanocomposite fabrication processes, such as template-directed CVD and ice freeze casting methods for fabricating porous structures, are very complex and costly. However, the nanocomposite manufacturing process of flexible conductive sponge triboelectric nanogenerators is simple and inexpensive. In the tribo-negative CNT/silicone rubber nanocomposite, the CNTs act as electrodes, increasing the contact area between the two triboelectric materials, increasing the charge density, and improving charge transfer between the two phases. Measurements of the performance of flexible conductive sponge triboelectric nanogenerators using an oscilloscope and a linear motor, under a driving force of 2–7 N, show that it generates an output voltage of up to 1120 V and a current of 25.6 µA. In addition, by using different weight percentages of carbon nanotubes (CNTs), it is shown that the output power increases with the weight percentage of carbon nanotubes (CNTs). The flexible conductive sponge triboelectric nanogenerator not only exhibits good performance and mechanical robustness but can also be directly used in light-emitting diodes connected in series. Furthermore, its output remains extremely stable even after 1000 bending cycles in an ambient environment. In sum, the results demonstrate that flexible conductive sponge triboelectric nanogenerators can effectively power small electronics and contribute to large-scale energy harvesting.

## 1. Introduction

With the rapid development of portable and wearable devices, the demand for self-powered devices has increased. Moreover, portable devices such as smartwatches and artificial intelligence robots connect to a network. Thus, considering the long-term and self-sustainable operation [1,2,3,4] of self-power-generating devices based on energy harvesting is attracting significant attention [5,6]. Energy harvesting technology is a process in which operational energy is derived from ambient energy, such as thermal, wind, and vibration energy [7,8]. Accordingly, several studies have researched energy-harvesting devices such as piezoelectric, biomechanical, electromagnetic, and triboelectric nanogenerators [9,10,11,12]. Among these, triboelectric nanogenerators (TENGs), based on the contact electrification and electrostatic effects, have the advantages of simple fabrication, excellent reliability, high efficiency, and low cost [13,14,15,16]. Their operational mechanism is based on the electron flow achieved by the triboelectric effect between two different triboelectric materials, which yields a surface charge transfer with periodic contact separation. In that way, TENGs can continuously and effectively generate electrical energy from even low-frequency energies, such as that generated by human motion [17,18,19].

Meanwhile, recent advances in polymer-based nanocomposites have made them more compatible as materials for TENGs in portable and wearable devices. Three-dimensional foam-shaped nanocomposites, for instance, have a large surface area, low density, porosity, and flexibility [20,21,22,23,24,25]. Several studies have analyzed nanocomposites using a combination of viscoelastic polymers and conductive nanomaterials such as carbon nanotubes (CNTs), graphene [26], metal nanoparticles [27], nanowires [28], and their hybrids [29]. CNTs are effective triboelectric polymer materials owing to their light weight, extremely high aspect ratio, good chemical stability, and thermal conductivity. Similarly, silicone rubber is widely used in wearable devices because it is flexible, non-flammable, and non-toxic. Therefore, a lot of research on TENGs for devices that are flexible, wearable, and self-powered has involved the use of CNT/silicone rubber nanocomposites [30,31,32]. However, nanocomposite fabrication processes, such as the template-directed CVD [33] and ice-freezing casting [34] methods, are highly complicated and expensive [35]. Therefore, the casting method using a sugar template, which is safe, stable, and easy to conduct, is used in our study.

Herein, we propose a flexible sponge-type TENG using a CNT/silicone rubber nanocomposite that can be fabricated using a simple casting method. This porous CNT/silicone rubber nanocomposite structure was characterized using SEM and Raman spectroscopy, and the output voltage and current were measured using a linear motor in the contact separation electrode mode. Furthermore, we examined the effect of increasing the force of the linear motor, and fabricated CNT/silicone rubber nanocomposites with different weight percentages of CNTs to observe the output performance. Additionally, we demonstrated that several light-emitting diodes (LEDs) can be powered by the flexible conductive sponge triboelectric nanogenerator (FCS-TENG). Finally, we examined the stability of the output performance after 1000 cycles of pushing and bending the proposed sample to confirm stable power generation. The results of our experiments show that the FCS-TENG has significant potential for application in flexible and self-powered electronic devices.

## 2. Materials and Methods

### 2.1. Materials

The silicone rubber (Dragon Skin NV10) used in this study was purchased commercially. Multiwalled carbon nanotubes (MWCNTs) were purchased from Jeio Co. The average diameter of the multiwalled carbon nanotubes (MWCNTs) is 10–15 nm and the length is 30–40 µm. Crystal sugars (average particle/grain diameter of 300 µm) were purchased from the market. All the chemicals were used without pretreatment.

### 2.2. Fabrication of FCS-TENG

Porous CNT/silicone rubber nanocomposites were formed by the templating technique, which is cheap, simple, and environmentally friendly, using the following sequence: create sugar cube templates on Petri dishes according to the weight percentage of CNTs (1.2, 2.4, 2.8, and 3.2 wt%), dry the sugar cube templates for 5 h at room temperature, immerse the sugar cube templates in silicone rubber solution that was prepared by mixing a base and a curing agent at a weight ratio of 1:1, cure the silicone rubber solution at room for 2 h, remove the sugar by immersing the samples in water for 1 h, dry the samples overnight, apply 0.3 mm thick silicone rubber on the surface, and finally, dry for 1 h.

### 2.3. Characterization of CNT/Silicone Rubber Nanocomposite

The CNT sponge structures were characterized using normal scanning electron microscopy (SEM) (JEOL Ltd., Akishima, Japan) (JSM-6010LA) to observe the morphology of the CNT/silicone rubber nanocomposite. A Raman spectrometer (NTEGRA Spectra) with a 633 nm laser as the excitation source was used to observe the crystallinity of the CNTs.

### 2.4. Evaluation of FCS-TENG

To measure the electrical output characteristics of FCS-TENG, a linear motor (Linmot, custom-made) to create a force of 2–7 N, an oscilloscope (TBS 2202 B, Tektronix, OR, USA), a current amplifier (DLPCA-200, Femto, Berlin, Germany), a high-voltage probe (P5100A, Tektronix, OR, USA), a bending tester (PMC-1HS/2HS, Autonics, Busan, Republic of Korea), and a control PC were used in the experimental setup. The linear motor was controlled by a control PC using Linmot software.

## 3. Results and Discussion

### 3.1. Principles for the Preparation of the FCS-TENG

**Figure 1:**
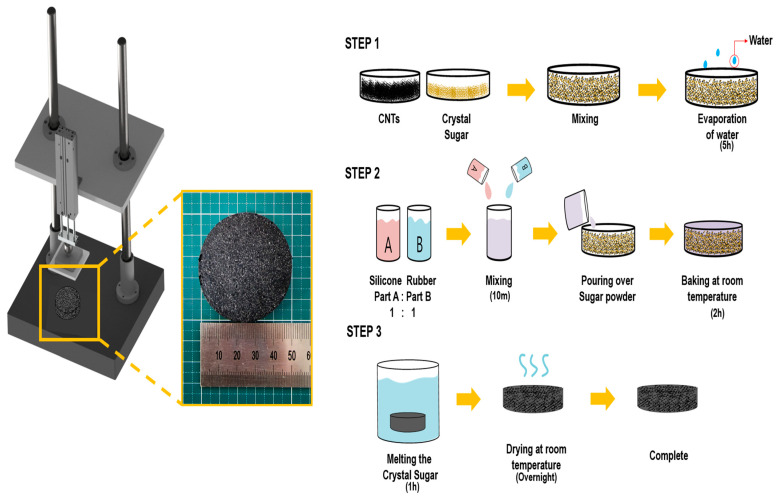
Manufacturing the FCS-TENG.

**Figure 2:**
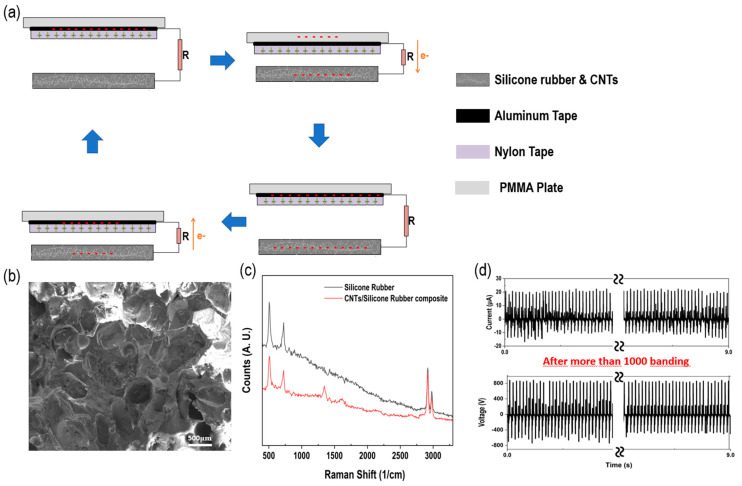
Schematic and features of FCS-TENG. (**a**) Schematic of the contact- and separation-mode mechanism. (**b**) Normal scanning electron microscope (Normal−SEM) image of flexible conductive sponge of FSC-TENG. (**c**) Raman spectra comparing silicon rubber with FCS−TENG (3.2 wt%). (**d**) Comparison of initial experimental values using a bending tester and experimental values after bending more than 1000 times.

The detailed manufacturing method of the TENG is shown in Figure 1, and detailed information can be found in the Materials and Manufacturing section. Information on the tensile force and flexibility, which are characteristics of the FCS-TENG, can be found in Figure S1. The operation method and schematic of the conductive FCS-TENG fabricated using CNT/silicone rubber nanocomposites are shown in Figure 2a. Figure 2 shows the current generation mechanism through charge transfer when the TENG operates in the contact and separation mode. As shown in Figure 2a, charge transfer does not occur because electrical neutrality is maintained in the initial state of complete separation or complete contact. However, in the process of contact or separation between the negatively charged flexible conductive sponge and the positively charged nylon, charge transfer occurs and electrical neutrality is achieved. During this time, the flexible conductive sponge becomes negatively charged with the property of easily gaining electrons, while nylon and aluminum become positively charged with the property of easily losing electrons. Since the polarities of the two materials are opposite, charge transfer occurs through the external circuit, resulting in electrical neutrality and current generation. Figure 2b shows a SEM image of the porous CNT/silicone rubber nanocomposite surface with an average pore size of 300 µm. In Figure S2, SEM images of porous CNT/silicone rubber nanocomposite surfaces at various magnifications can be found. The Raman spectra of the silicone rubber and CNT/silicone rubber composites are shown in Figure 2c. The Raman analysis clearly explains the presence of CNTs. The observed characteristic peaks of silicon rubber are as follows: the peak at 504 cm^−1^ corresponds to the existence of Si-o-Si, and the 725 cm^−1^ peak reveals the existence of the stretching vibration of Si-(CH_3_)_2_. The peaks at 2917 cm^−1^ and 2977 cm^−1^ correspond to the stretching vibration of the methyl (-CH_3_) group [36,37,38]. In contrast, the presence of CNTs (when 3.2 wt% of MWCNT is mixed with silicone rubber) in silicone rubber is revealed by the characteristic peak at 1344 cm^−1^, which signifies the presence of a disorder-induced band (D-band). In addition, the other peaks are consistent with the corresponding observations of previous studies [39,40,41]. The bending stability of the FCS-TENG’s output performance is illustrated in Figure 2d, which is crucial for determining the possible applications of TENGs, such as in flexible devices. To confirm the output stability, the produced FCS-TENG (2.8 wt%) was subjected to more than 1000 bending tests. The operating motion and distance for the bending test are shown in Figure S4. The initial and final outputs of the FCS-TENG were 880 V, 20.8 μA, and 880 V, 22.4 μA, respectively, indicating consistent output even after 1000 bending cycles. Therefore, we conclude that the FCS-TENG is highly flexible, robust, and durable, and is suitable for various curved device applications.

### 3.2. Electrical Performance of the FCS-TENG

**Figure 3:**
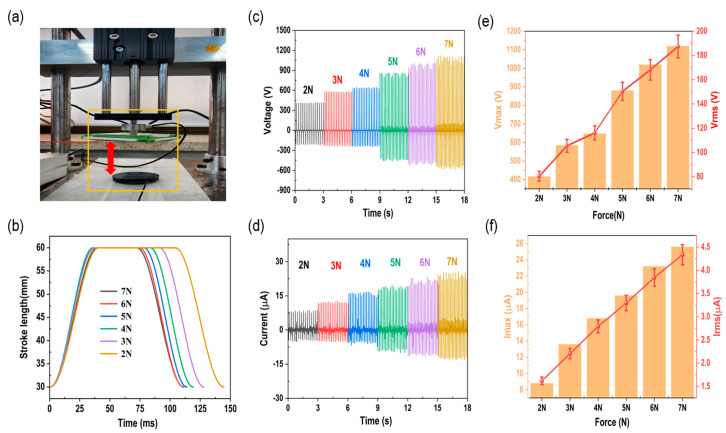
Output performance evaluation by adjusting stroke duration and stroke length. (**a**) Linear motor expressing reciprocating motion, (**b**) measurement graph of linear motor stroke duration, (**c**,**d**) voltage and current output graph from 2 to 7 N, and (**e**,**f**) maximum and RMS values from 2 to 7 N.

Figure 3a a shows the optimal electrical output characteristics when the TENG operates in the vertical contact separation mode. Through this experiment, the highest output under the conditions applied to the FCS-TENG (2.8 wt%) device was confirmed. As shown in Figure 3b, the stroke distance and stroke duration of the liner motor were adjusted to enable us to control the force from 2 to 7 N using the PC. As the distance between the start and end points of a stroke shortens, the acceleration increases and the applied force increases. Therefore, by changing the distance of the stroke, the duration of the stroke was controlled to control the force from 2 to 7 N. Corresponding to the driving force range between 2 and 7 N in Figure 3c,d, the output in Figure 3e,f is 1120 V, 25.6 μA, and the minimum output is 416 V, 8.8 μA. Table 1 summarizes the output values according to the change in force. The output performance of the TENG can be expressed by the following governing equation [41,42].

(1) $$\Delta V=-\frac{Q}{S\varepsilon_{0}}\left(\frac{d_{1}}{\varepsilon_{r1}}+x\left(t\right)\right)+\frac{\sigma x\left(t\right)}{\varepsilon_{0}},$$

(2) $$I=-\frac{S\left(t\right)\sigma d}{\varepsilon_{r}{\left(\frac{d}{\varepsilon_{r}}+x\left(t\right)\right)}^{2}}\cdot \frac{dx\left(t\right)}{dt},$$

where Q is the value of the transferred charges between the two electrodes, S is the dielectric area size, $\varepsilon_{0}$ is the vacuum permittivity, εr is the relative permittivity, σ is the friction charge surface density, $x\left(t\right)$ is the distance between the two contact surfaces, t is the time, and d is the effective dielectric thickness. As can be seen from Equations (1) and (2), when the contact area increases, both the output voltage and current increase. In other words, as the contact area between the FCS-TENG, which is the negative charge, and the nylon tape, which is the positive charge, increases, and the stroke length shortens, the linear motor plate can apply a greater force on the TENG. Furthermore, the FCS-TENG can show a high output signal even with a fast response speed, which is highly compatible with various signal sensor applications.

**Figure 4:**
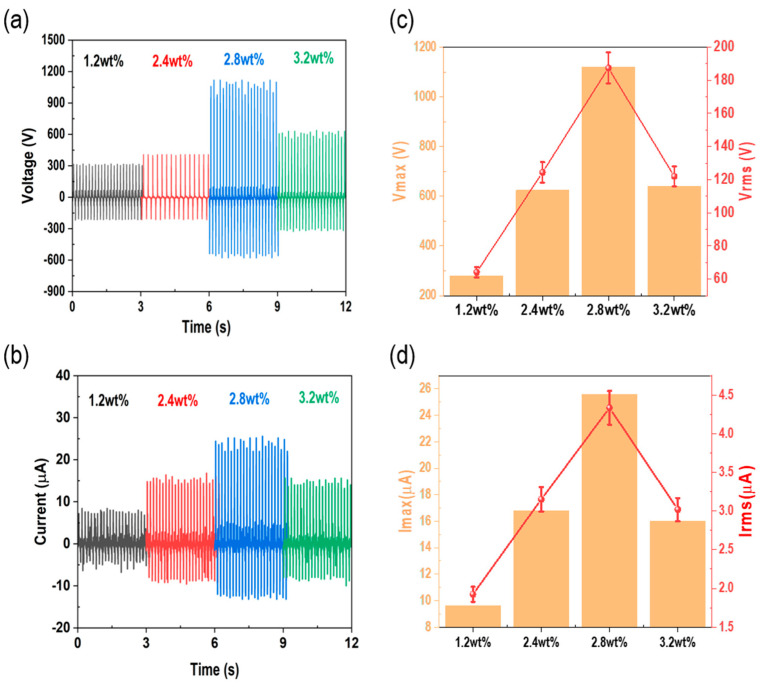
Evaluation of FCS-TENG output performance according to CNT ratio. (**a**,**b**) Voltage and current output graph from 1.2 to 3.2 wt%, and (**c**,**d**) maximum value and RMS value graph from 1.2 to 3.2 wt%.

The Raman spectra results for different weight ratios of CNTs (1.2 wt%, 2.4 wt%, 2.8 wt%, and 3.2 wt%) in the flexible conductive sponge TENG (FCS-TENG) are presented in Figure S3. Subsequently, FCS-TENGs were fabricated with these weight ratios to investigate their effects on TENG performance. As shown in Figure 4, the output of the FCS-TENG increases as the weight percentage of CNTs increases, before decreasing at 3.2 wt%. Clearly, the amount of mixing varies according to the ratio of silicone rubber and CNTs. Moreover, at higher ratios, the silicone rubber is harder and the degree of compression is lower, which results in a decrease in the output. At a driving force of 7 N, the voltage and current increase from 416 V to 1120 V and 8.8 to 25.6 μA, respectively. The increase in CNTs leads to the formation of a more efficient compound for charge transport, which enhances the conductivity of the polymer. Therefore, since the increasing trends of e and f in Figure 3 increase linearly with the change in force, the maximum output value can be expressed by varying the ratio of CNTs [42,43,44]. The FSC-TENG (2.8 wt%) shown in Figure 4c,d has a high-power source capable of capturing various forms of environmental energy. Therefore, it can be used in a variety of functions.

### 3.3. Application

**Figure 5:**
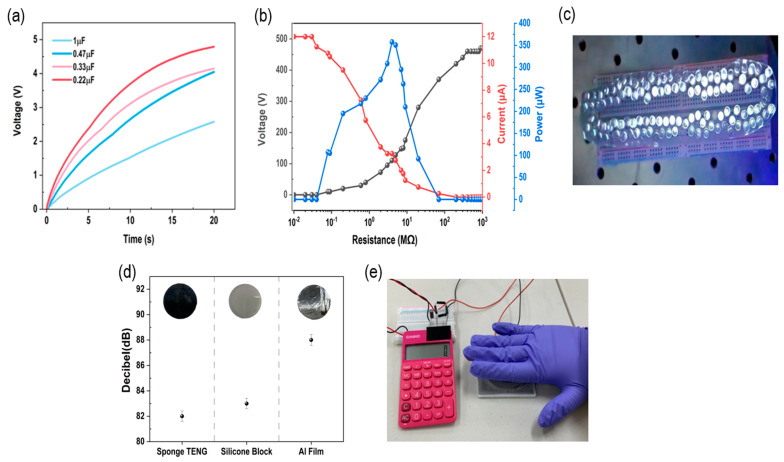
Performance check using FCS-TENG. (**a**) Graph depicting the use of capacitors with different capacitance values (0.22, 0.33, 0.47, 1 μF), (**b**) output performance test at varying external impedance levels, (**c**) 118 white LED lights arranged in a row, (**d**) noise test conducted with three materials (FCS−TENG, silicone block, Al film), and (**e**) calculator-charging test using FCS−TENG.

The FCS-TENG generates power from the alternating current (AC). Figure 5a shows that the 1 μF capacitor is charged up to 2 V in 17 s using a bridge rectifier circuit that converts AC to direct current (DC). To confirm the applicability of the FCS-TENG, a charging test is conducted for capacitors with various capacitances (0.22, 0.33, 0.47, and 1 μF) using a bridge-rectifier circuit. The 2.8 wt% FCS-TENG is applied with a controlled force of 7 N for the experiment. As shown in Figure 5a,b, the measured voltage increases over time. Subsequently, to check the power generated in the FCS-TENG shown in Figure 5b, it is measured at a variable resistance of between 0.1 and 1 GΩ, and the output power value is calculated for each external resistance value; the maximum power value is 375.5 μW at 40 MΩ. Figure 5c shows the LED test setup. In this test, the power generation performance of the FCS-TENG is confirmed by turning on 118 white LEDs, which provide output values identical to those from the experiments shown in Figure 5a, b. In this manner, the power generation and charging performance of the FSC-TENG are verified. Subsequently, the sponge-type half of FCS-TENG, TENG composed of film, and TENG composed of silicone rubber are subjected to a noise test. The lowest decibel value is obtained in the case of FCS-TENG, which can be attributed to its porous structure, which absorbs sound and acts as a noise barrier. Finally, the electric energy stored in the capacitor charged using the bridge rectifier circuit is used as the power source for a calculator, as shown in Figure 5e. These results indicate that the FCS-TENG can be used as a sustainable power source capable of providing sufficient power to portable electronic devices. Furthermore, we successfully used the FCS-TENG directly, without the aid of an external power source, to power a portable self-powered device, which confirms the great potential of TENGs in electronic devices.

## 4. Conclusions

In summary, the FCS-TENG proposed in this study consists of a highly flexible and stretchable sponge structure with embedded CNTs that can power small electronic devices anywhere. In particular, it is possible to fabricate an efficient porous structure with an easy fabrication method and low cost. When a force of 7 N was applied to a 2.8 wt% FCS-TENG, an electrical output characterized by a voltage of up to 1120 V and a current of 25.6 μA was obtained. Furthermore, the electrical output of the FCS-TENG was used to power 118 white LEDs connected in series to demonstrate the practical capabilities of the generator, and was used to operate portable small electronic devices to evaluate its self-generation efficiency. Through repeated bending experiments, we believe that FCS-TENG is very flexible, strong and durable, so it can be applied to various curved devices. Consequently, the results presented herein give evidence of the excellent output performance of the FCS-TENG, which means it can be used for low-power IoT devices, wearable monitoring systems, and flexible sensors. It can also be conveniently used regardless of the place or environment thanks to its modular composition.

## Figures and Tables

**Table 1:** Output value from 2 to 7 N of flexible conductive sponge triboelectric nanogenerators (2.8 wt%).

	Vmax	Vrms	Imax	Irms
**2 N**	416 V	80.3603 V	8.8 μA	1.6179 μA
**3 N**	584 V	105.468 V	13.6 μA	2.2061 μA
**4 N**	648 V	116.146 V	16.8 μA	2.791 μA
**5 N**	880 V	150.401 V	19.6 μA	3.2949 μA
**6 N**	1020 V	168.165 V	23.2 μA	3.8435 μA
**7 N**	1120 V	187.434 V	25.6 μA	4.3349 μA

## Data Availability

The data are available on reasonable request from the corresponding author.

## Supplementary Materials

The following supporting information can be downloaded at: https://www.mdpi.com/article/10.3390/polym15051135/s1, Figure S1: Confirmation of FCS-TENG (2.8 wt%) tensile force and flexibility; Figure S2: Normal scanning electron microscope (Normal-SEM) image according to magnification change of flexible conductive sponge TENG; Figure S3: Raman spectra according to the CNT ratio (1.2, 2.4, 2.8, and 3.2 wt%) of FCS-TENG; Figure S4: Bending tester operating motion and distance; Video S1: We turned on 118 white LEDs to check the output performance of the FCS-TENG; Video S2: Power supply to the portable calculator after charging the capacitor using the bridge rectifier circuit.
