# Supplementary material for: Highly Flexible Triboelectric Nanogenerator Using Porous Carbon Nanotube Composites
Source: Polymers (Basel). 2023 Feb 24;15(5):1135. doi: 10.3390/polym15051135 (PMC10006981; doi:10.3390/polym15051135)
Supplement: Supplementary file 1 [file polymers-15-01135-s001.zip › Supplementary_Information.pdf]

Supplementary Information

Highly Flexible Triboelectric Nanogenerator Using Porous Carbon Nanotube Composites

Jaehee Shin [1], Sungho Ji[1], Hanchul Cho[2,*] and Jinhyoung Park [1,*]

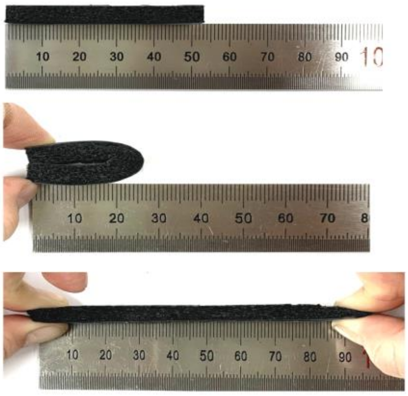

Figure S1. Confirmation of FCS-TENG (2.8wt%) tensile force and flexibility.

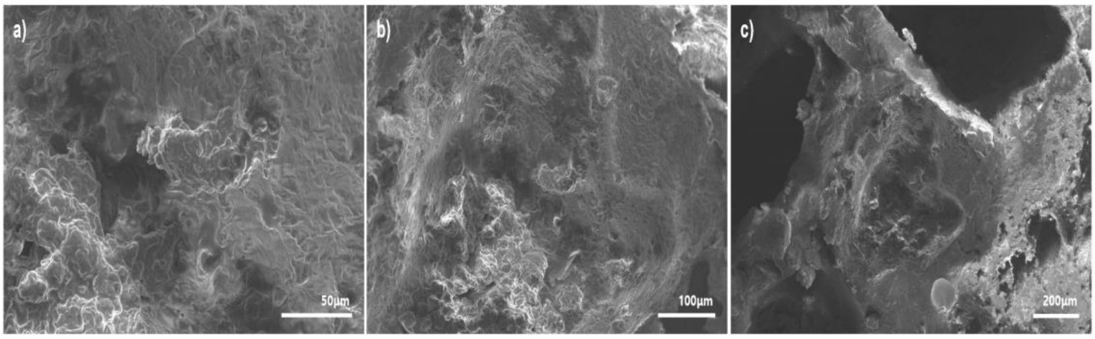

Figure S2. Normal scanning electron microscope (Normal-SEM) image according to magnification change of flexible conductive sponge TENG. (a) The scale bar is 50 μm. (b) The scale bar is 100 μm. (c) The scale bar is 200 μm.

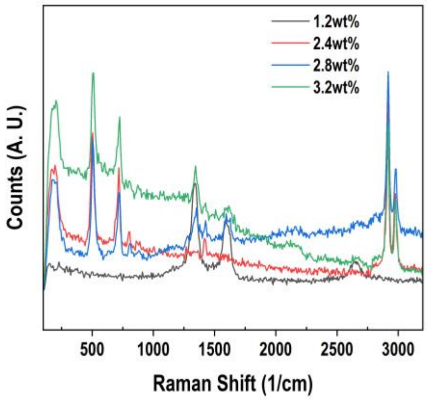

Figure S3. Raman spectra according to CNTs ratio (1.2wt%, 2.4wt%, 2.8wt%, 3.2wt%) of Flexible Conductive Sponge TENG(FCS-TENG).

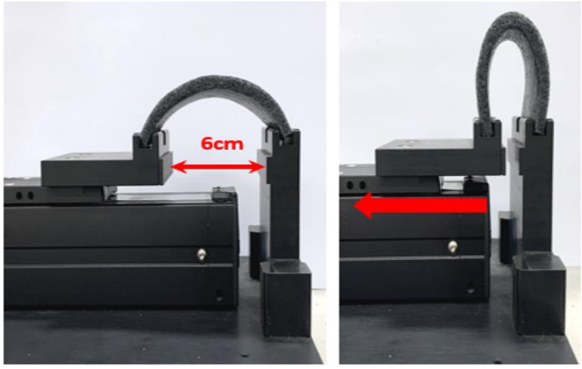

Figure S4. Bending tester operating motion and distance.
